# Frequency of CD4^+^ and CD8^+^ T cells in Iranian chronic rhinosinusitis patients

**DOI:** 10.1186/s13223-018-0270-9

**Published:** 2018-07-05

**Authors:** Farhad Seif, Babak Ghalehbaghi, Hossein Aazami, Alireza Mohebbi, Aslan Ahmadi, Reza Falak, Pegah Babaheidarian, Mohammad Najafi, Majid Khoshmirsafa, Sahand Ghalehbaghi, Mehdi Shekarabi

**Affiliations:** 1 Department of Immunology, School of Medicine, Iran University of Medical Sciences, Tehran, Iran; 2 ENT and Head and Neck Research Center and Department, Hazrat Rasoul Akram Hospital, Iran University of Medical Sciences, Tehran, Iran; 3 Immunology Research Center, Institute of Immunology and Infectious Diseases, Iran University of Medical Sciences, Tehran, Iran; 4 Department of Pathology, Rasoul Akram Medical Complex, Iran University of Medical Sciences, Tehran, Iran; 5 Department of Biochemistry, School of Medicine, Iran University of Medical Sciences, Tehran, Iran

**Keywords:** Chronic rhinosinusitis, CRSsNP, CRSwNP, T cell, CD4, CD8, Macrophage

## Abstract

**Background:**

Chronic Rhinosinusitis (CRS) is a persistent inflammatory disease affecting paranasal sinuses. CRS is categorized into two distinct subgroups defined as CRS with nasal polyps (CRSwNP) and CRS without nasal polyps (CRSsNP). Although several immune cells are involved in the CRS pathogenesis, the role of T cells is not fully understood. The objective of the present study was to evaluate the frequency of CD4^+^ and CD8^+^ T cells and macrophages in the sinonasal mucosa of CRS patients, as well as to investigate the specific transcription factors for Th1, Th2, Th17, and Treg cells.

**Methods:**

In this study, 15 healthy controls, 12 CRSsNP, and 23 CRSwNP patients participated. CD4^+^, CD8^+^, and CD68^+^ cells were investigated in the sinonasal tissues using immunohistochemistry. The expression of transcription factors related to Th subsets (T-bet, GATA3, Ror-γt, and FoxP3) was evaluated using real-time PCR. Furthermore, CRSwNP patients were defined as eosinophilic when eosinophils consisted of more than 10% of total inflammatory cells. The Kruskal–Wallis, Mann–Whitney, and Spearman tests were used in statistical analyses.

**Results:**

The median (range) age of the studied groups was: 32 (14–67) for CRSwNP, 28 (10–43) for CRSsNP, and 27 (17–44) for controls. The number of eosinophils in CRSwNP patients was higher than two other groups, whereas neutrophils were elevated in both CRSwNP and CRSsNP groups in comparison to controls. The frequency of CD4^+^ and CD8^+^ T cells, macrophages, and total inflammatory cells were significantly increased in CRSwNP and CRSsNP patients compared with controls. The mRNA expression of GATA3 was increased in CRSwNP patients while mRNA expression of Ror-γt was elevated in CRSsNP patients. No significant difference was observed in T-bet mRNA expression among three groups. Both CRSwNP and CRSsNP patients showed decreased FoxP3 mRNA expression in comparison to controls.

**Conclusion:**

The frequency of CD4^+^ and CD8^+^ T cells was elevated in CRS patients. In addition, we demonstrated Th2 dominance in CRSwNP patients and Th17 dominance in CRSsNP patients, implicating different mechanisms may underlie the disease. Better CRS classification and targeted therapeutic strategies may be achievable by determining the pattern of infiltrating inflammatory cells. Therefore, further experimental investigations on T cells are needed.

## Background

Chronic rhinosinusitis (CRS) is a prevalent disease characterized by persistent inflammation of the paranasal sinuses and upper airway tracts for at least 12 weeks. With regard to either endoscopic or relevant CT observations, as well as clinical symptoms, CRS can be categorized into two distinct groups: CRS with nasal polyps (CRSwNP) and CRS without nasal polyps (CRSsNP) [1, 2]. CRS is associated with a prominent socioeconomic burden on public health worldwide. Recently, it is universally established that inflammatory mechanisms of these two subgroups are different [3]. Genetic factors, immune system, and nasal microbiome play important roles in the CRS development [4]. However, the exact etiology of CRS is uncertain and different immunological mechanisms have been described in the CRS pathogenesis [5]. Although several therapeutic approaches are suggested for CRS management, the disease treatment remains challenging [6, 7].

T lymphocytes play major roles in the regulation of inflammatory process at mucosal sites [8–10]. The remarkable roles of CD4^+^ Th (T helper) cells were partially determined in the pathogenesis of CRS. These cells consist of IFN-γ^+^ Th1, IL-4^+^ Th2, IL-17A^+^ Th17, and CD4^+^ regulatory T (Treg) cells [11, 12]. Previous studies showed that the CRS signature is Th1, Th2, and Th17 mixture in airway mucosa [8]. CD8^+^ T cells (cytotoxic or T_CTL_) are another population of T cells that cytolyze and eliminate tumor cells and the cells infected with intracellular pathogens. The occurrence of cytolysis is dependent on the perforin and granzyme B secretions to induce cell apoptosis in target cells [13]. Similar to CD4^+^ Th cells, CD8^+^ cytotoxic T cells (Tc) can be divided into several subsets, including IFN-γ^+^ Tc1, IL-4^+^ Tc2, IL-17A^+^ Tc17, and CD8^+^ regulatory T (Treg) cells [14, 15]. Previous studies that have also noted that Tc2 cells are involved in eosinophilic immune responses, whereas Tc1 and Tc17 cells have been shown to induce neutrophilic immune responses in inflammatory sites [15]. It has been demonstrated that CRSwNP is characterized by predominant Th2 responses, whereas CRSsNP is distinguished by elevated Th1 responses [16]. Although the roles of CD4^+^ T cells are extensively described in CRS, the roles of CD8^+^ T cells are poorly investigated. Therefore, the aim of the present study was to determine the frequency of infiltrating CD4^+^ and CD8^+^ T cells and macrophages (CD68^+^ cells), additionally the mRNA expression of Th cell subsets (T-bet, GATA3, Ror-γt, and FoxP3) in the sinonasal mucosa of CRSwNP and CRSsNP patients in comparison to healthy controls. We found that the frequency of CD4^+^ and CD8^+^ T cells were significantly elevated in CRS patients. In addition, we showed that the expression of GATA3 was increased in CRSwNP patients, whereas the expression of Ror-γt was elevated in CRSsNP patients. Finally, CRS patients indicated lower FoxP3 expression than controls.

## Patients and methods

### Patient selection

Patients suffering from CRS were recruited from the ENT and Head and Neck clinic at Hazrat Rasoul Akram Hospital, Iran University of Medical Sciences. Twenty-three patients with CRSwNP and 12 patients with CRSsNP enrolled in our study. Fifteen inferior turbinate samples were collected as controls from subjects undergoing septoplasty due only to nasal septum deviation without a history of CRS or asthma. The diagnosis of CRS was made according to the current European Position Paper on Rhinosinusitis and nasal polyps [17], fulfilling two or more of the following criteria: blockage/congestion/obstruction, nasal discharge, facial pain/pressure, decrease or loss of smell for at least 4 weeks. In addition, CT scan and endoscopic scores were recorded to confirm nasal polyp(s). Demographic characteristics of population study are summarized in Table 1. Lund-Kennedy nasal endoscopy scores [18], preoperative Lund-Mackay CT scores [19], as well as preoperative and postoperative 22-item Sinonasal Outcome Test (SNOT-22) [20], were recorded to calculate clinical scores of each CRS patient. This study was approved by the ethics committee of Iran University of Medical Sciences (IR.IUMS.REC 1395.95-03-30-27364) and all patients filled written informed consent for tissue sample collection. We performed ethical clearance according to the ethical standards of the relevant national and institutional guidelines on human experimentation and with the Helsinki Declaration of 1975, as revised in 2008. Exclusion criteria are as follow: CRS patients (1) with immunodeficiencies, cystic fibrosis, bronchiectasis, chronic obstructive pulmonary disease, diabetes mellitus, neoplasia, or fungal rhinosinusitis; (2) during pregnancy or lactation, and (3) with upper airway infections within 1 month ago. All the patients enrolled for surgery had previously failed to respond to adequate medical treatments. None of the subjects had used systemic or nasal corticosteroids, antibiotics, antihistamines, decongestants, and anti-leukotrienes 4 weeks before biopsy/surgery.

**Table 1:** Characteristics of controls and CRS patients

	Controls	CRSsNP	CRwNP	P value
Number	15	12	23	
Females/males	5/10	4/8	6/17	
Age (year) median (min–max)	27 (17–44)	28 (10–43)	32 (14–67)	> 0.05
Asthma	0	0	5	
CT score^a^	0	7.5 (3–10.75)	19 (14–21)	< 0.0001
Endoscopic score^a^	0	1 (0–1)	2 (2–3)	< 0.001
VAS score^a^	0	5 (4–7.750)	10 (9–11)	< 0.05

*VAS* visual analogue scales

^a^Data are expressed as median (IQR)

### Biopsy and specimens

Tissue samples were divided into three sections; the first section was stored at − 80 °C for subsequent RNA isolation and the second section was used for protein isolation. The last section was fixed overnight for immunohistochemistry in a freshly prepared fixative containing 4% paraformaldehyde in PBS (pH 7.4) and was embedded in paraffin wax.

### Histological analysis

We used Hematoxylin & Eosin (H&E) staining to evaluate the pathologic characteristic of the tissues. To this aim, paraffin sections (5 mm) were stained with H&E. Air-dried sections were stained with H&E for 60 min at room temperature (RT). Then, the stained sections were analyzed by a pathologist who was blind to the clinical data. The number of eosinophils, neutrophils, mononuclear cells, total inflammatory cells (eosinophils, neutrophils, T cells, macrophages and mononuclear cells), goblet cells, and mucosal glands in the lamina propria was counted at high power field (HPF) 400×, using an Olympus CX-40 microscope (Olympus, Tokyo, Japan) and 5 random HPFs were selected and observed. Results were presented as cells or glands per HPF [8]. CRSwNP patients were categorized as eosinophilic when eosinophils consisted of more than 10% of total inflammatory cells, and as non-eosinophilic when eosinophils consisted of less than 10% of the total inflammatory cells [8].

### Immunohistochemistry

CD4 (a marker representing Th cells), CD8 (a marker representing CD8^+^ T cells), and CD68 (a marker representing macrophages) were evaluated by immu-nohistochemistry. Sinonasal tissues were dehydrated and embedded in the paraffin. Tissue samples were sectioned at 3 µm. Then, they were rehydrated through a xylene and ethanol series and were immersed in Target Retrieval solution (low pH, Dako, Glostrup, Denmark) and autoclaved at 121 °C for 20 min for the retrieval of antigens. Endogenous peroxidase activity was blocked with 3% H2O2/methanol. After washing, sections were incubated for 30 min in a blocking solution (PBS, pH 7.4, containing 2% bovine serum albumin [Sigma-Aldrich, Darmstadt, Germany], 0.1% Triton X-100, and 0.1% sodium azide) at RT to decrease nonspecific bindings [21], then incubated with: Monoclonal Mouse Anti-Human CD4 (1:100, Clone 4B12, Dako), Monoclonal Mouse Anti-Human CD8 (1:200, Clone C8/144B, Dako), Monoclonal Mouse Anti-Human CD68 (1:100, Clone KP1, Dako) for 1 h at RT. After the incubation process, all slides were washed with Tris-buffered saline (TBS) for 10 min and incubated for another 45 min at 30 °C with EnVision™ (Dako), using an Autostainer (Dako). The samples were counterstained with Mayer’s hematoxylin stain and mounted in Faramount Mounting Medium (Dako), prior to analysis by light micros-copy. The number of positive cells in tissue sections was counted by a light microscope at a magnification of 400×, using an Olympus CX-40 microscope (Olympus, Tokyo, Japan) [22].

### Quantitative real-time polymerase chain reaction

Total RNA was isolated from sinonasal tissues with Trizol (Invitrogen, USA) according to the manufacturer’s instructions. RNA integrity and the success of the reverse transcription reaction were monitored by PCR amplification of glyceraldehyde-3-phosphate dehydrogenase transcripts and denaturing agarose gel 2%. Genomic DNA was removed from total RNA, using RNase-free DNase Set (Qiagen, Chatsworth, CA, USA). Then, 500 ng of total RNA from each sample was utilized to prepare cDNA, using PrimeScript™ RT reagent Kit (TaKaRa, Korea). Reverse transcription reaction was done in total 20 µL of a reaction mixture containing 2.5 U of MML-V (RT; GIBCO BRL, Grand Island, NY) and 50 pmol of random hexanucleotides at 42 °C for 60 min, followed by 85 °C for 5 s to inactivate reverse transcriptase enzyme. Each real-time PCR reaction was carried out in a final volume of 20 µL, including 10 µL of 2 × SYBR Green Real-time PCR Master Mix (TaKaRa, Korea), 1 µL of cDNA, and 1 µL of the forward and reverse 200 nM primers. Instead of cDNA, nuclease-free water was added to each negative control microtube. The mRNA expression levels of specific transcription factors for Th1 (T-bet), Th2 (GATA3), Th17 (Ror-γt), and Treg (FoxP3) cells were subsequently determined using extracted RNA from tissue biopsies using quantitative real-time PCR. The primer sequences are listed in Table 2. Real-time PCR was performed using Rotor-Gene Q (Qiagen, Hilden, Germany). Conditions for 40 cycles of PCR were 95 °C denaturation for 5 s, 60 °C annealing-extension for 30 Sec. Experiments were assayed in duplicate for each sample. The mean threshold cycle values were normalized to beta actin (β-actin) expression levels, and the relative mRNA levels of target genes were calculated by 2^−ΔΔCt^ method [23, 24].

**Table 2:** Primer sequences used in real-time PCR

Gene	Size (bp)	Forward and reverse primers
T-bet	115	F: 5′- CTGGAGGTGTCGGGGAAAC-3′
		R: 5′-ATGGGAACATCCGCCGTCC-3′
GATA-3	107	F: 5′-TCATTAAGCCCAAGCGAAGG-3′
		R: 5′-GTCCCCATTGGCATTCCTC-3′
Ror-γt	144	F: 5′- AGACTCATCGCCAAAGCA -3′
		R: 5′- CCTTGTAGAGTGGAGGGAAA -3′
FoxP-3	124	F: 5′-ATTCCCAGAGTTCCTCCACAAC-3′
		R: 5′-ATTGAGTGTCCGCTGCTTCTC-3′
β-Actin	131	F: 5′-TCCCTGGAGAAGAGCTACG-3′
		R: 5′-GTAGTTTCGTGGATGCCACA-3′

### Statistical analyses

Statistical analyses were carried out with SPSS version 23.0 (SPSS Inc., Chicago, Illinois, USA) and presented using GraphPad Prism software version 6.1 (GraphPad, La Jolla, California). The normality of the data was evaluated by Kolmogorov–Smirnov test. The Kruskal–Wallis H test was used to assess significant intergroup variations. The Mann–Whitney U two-tailed test was used for between-group comparisons. The Spearman test was used to determine correlations between cellular frequencies. A value of *P* less than 0.05 was considered statistically significant.

## Results

### Comparison of the subject characteristics

In this study, 23 patients (6 females and 17 males) with CRSwNP, 12 patients with CRSsNP (4 females and 8 males), and 15 healthy controls (5 females and 10 males) were evaluated. The median (min–max) age of the population study was as follows: 32 (14–67) for CRSwNP, 28 (10–43) for CRSsNP, and 27 (17–44) for controls. We also evaluated and compared CT score, endoscopic score, and the VAS score in CRS patients, demonstrating sinus involvement in comparison to age- and sex-matched controls. There was a significant difference between CRSwNP and CRSsNP patients in CT score (< 0.0001), endoscopic score (< 0.001), and the VAS score (< 0.05). Five patients in CRSwNP group had asthma who were diagnosed according to the history. Approximately all CRSwNP patients with concomitant asthma represented high eosinophil infiltration. The demographic and clinical characteristics of CRS patients and controls are shown in Table 1.

### Eosinophils were elevated in CRSwNP patients

**Fig. 1:**
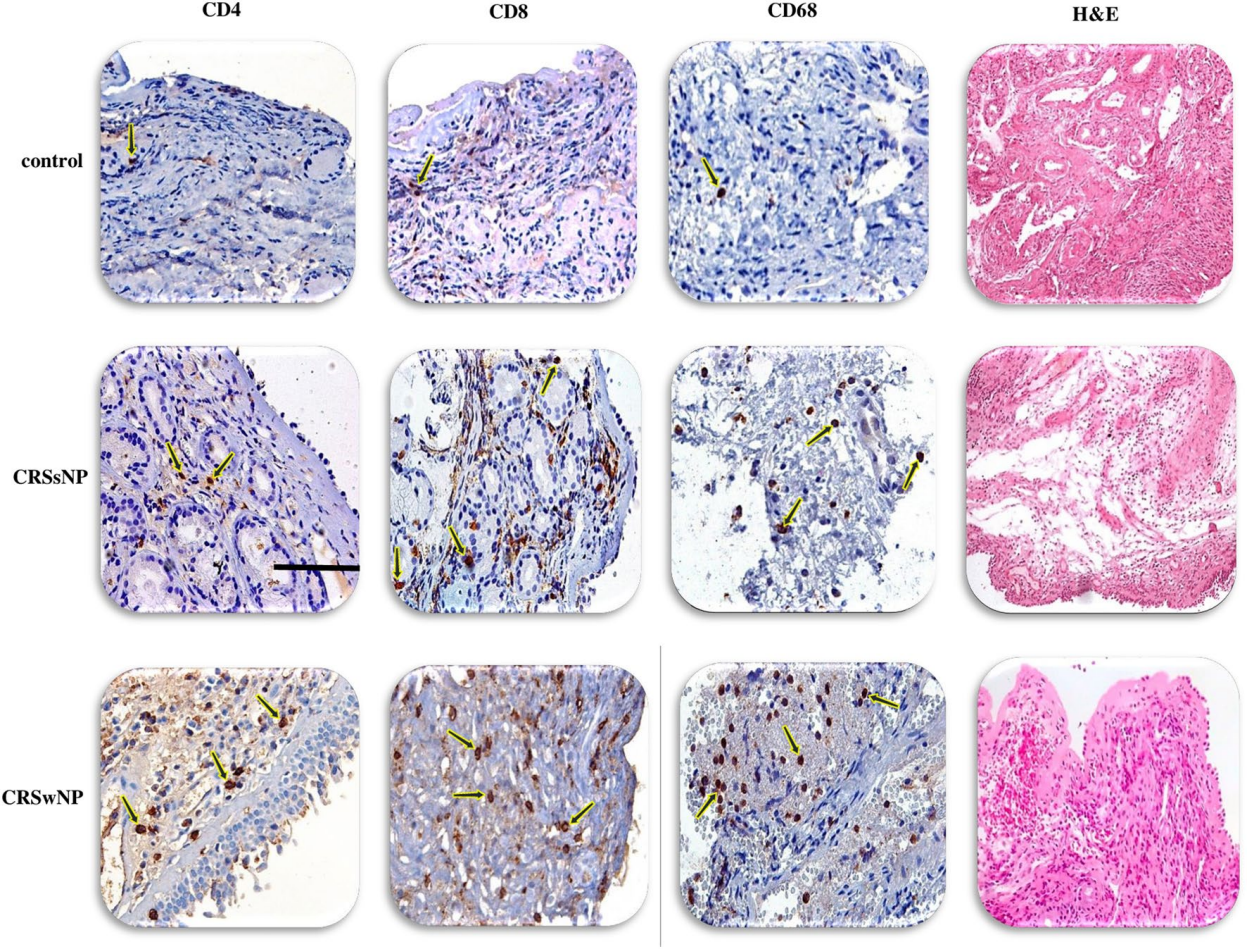
The distribution of infiltrating inflammatory cells to the sinonasal tissues. Representative IHC staining of CD4^+^ T cells, CD8^+^ T cells, and CD68^+^ macrophages, as well as H&E staining for eosinophils, neutrophils, and total inflammatory cells in controls, CRSsNP, and CRSwNP patients. Scale bar 100 µm. *CRSsNP* chronic rhinosinusitis without polyp, *CRSwNP* chronic rhinosinusitis with polyp

**Fig. 2:**
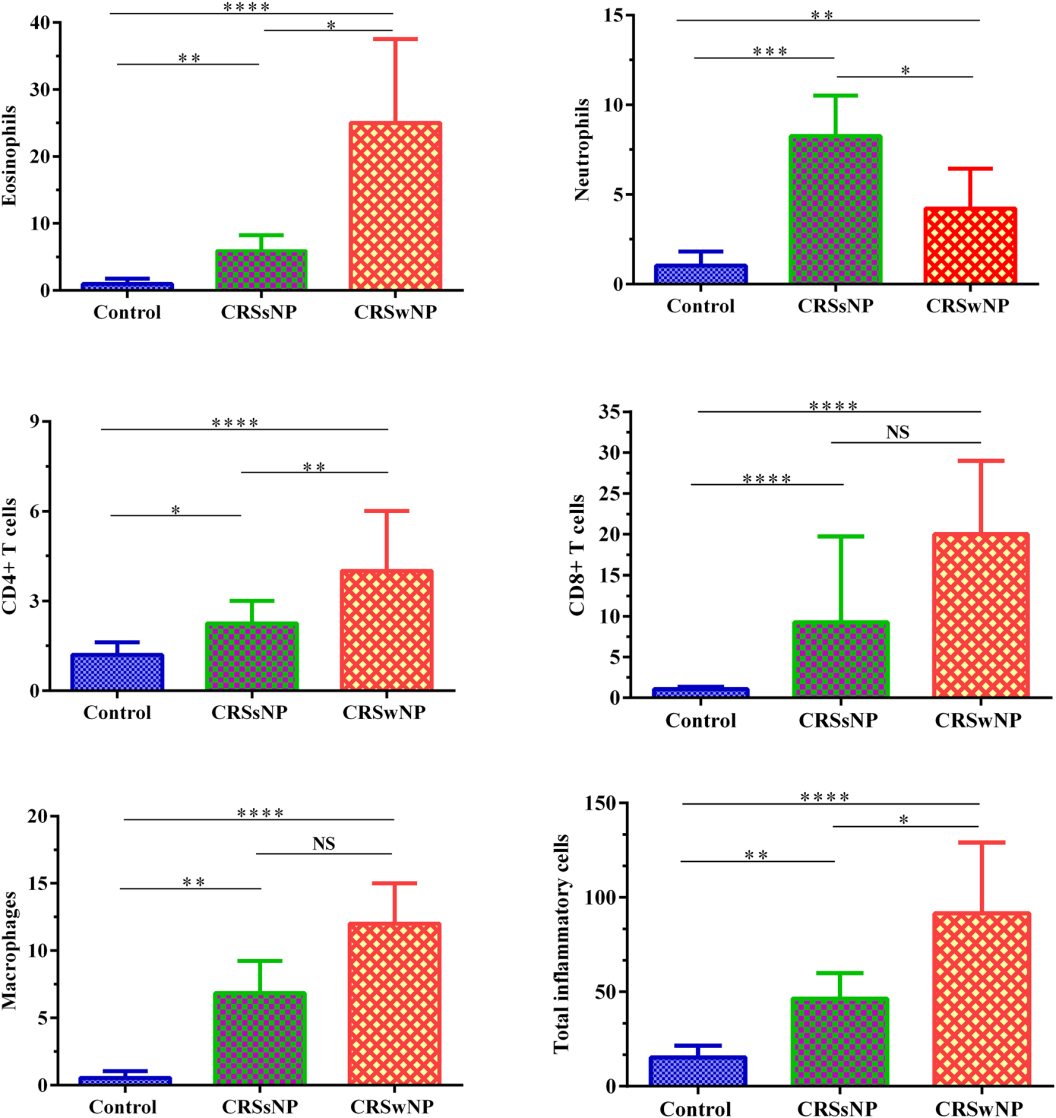
The mean numbers of eosinophils, neutrophils, CD4^+^ T cells, CD8^+^ T cells, macrophages, and total inflammatory cells in controls, CRSsNP, and CRSwNP patients. Eosinophils and CD4^+^ T cells were elevated in CRSwNP patients and neutrophils in CRSsNP patients. CD8^+^ T cells and macrophages were increased in both CRSwNP and CRSsNP groups. The Kruskal–Wallis H test was used to assess significant intergroup variability. *Pv* < 0.05 was considered as significant results

**Fig. 3:**
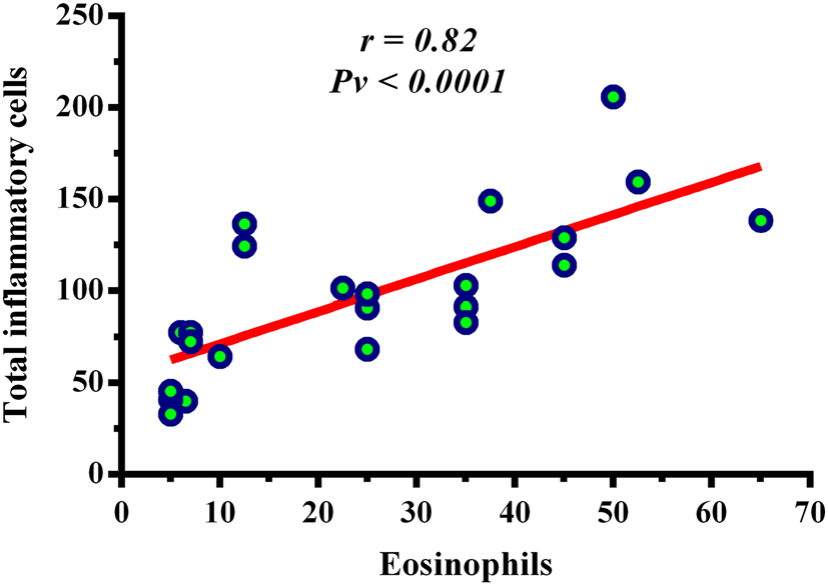
Correlation between the number of eosinophils and total inflammatory cells in CRSwNP patients. There was a significant correlation between eosinophils and total inflammatory cells in this group (r = 0.82, Pv < 0.0001). The Spearman test was used to determine the correlation

In this study, H&E findings showed that CRSwNP group was eosinophil dominant (Fig. 1, Table 3) whereas CRSsNP group was neutrophil dominant (Fig. 1, Table 3). The number of eosinophils was higher in CRSwNP patients than CRSsNP patients and controls (CRSwNP vs. CRSsNP vs. controls: *Pv* < 0.0001 for CRSwNP vs. controls, *Pv* = 0.003 for CRSsNP vs. controls, and *Pv* = 0.013 for CRSsNP vs. CRSwNP) (Fig. 2, Table 3). It was only observed that the number of eosinophils positively correlated with the number of total inflammatory cells (*r *= 0.82, *Pv *< 0.0001), but no relationship was found between eosinophils and other cells (Fig. 3). The number of total inflammatory cells in CRSwNP, CRSsNP, and control groups was 91.5 (68.5–129), 42.25 (34–54.5), and 12.5 (10–20), respectively (Fig. 1, Table 3). According to the proportion of eosinophils to the total inflammatory cells, we divided CRSwNP patients into 16 eosinophilic CRSwNP (ECRS) patients and 7 non-eosinophilic CRSwNP (N-ECRS) patients (Table 4).

**Table 3:** Comparison of Immunohistochemistry and real-time PCR results in controls and CRS patients

	Control	CRSsNP	CRSwNP	Control vs CRSsNP P value	Control vs CRSwNP P value	CRSwNP vs CRSsNP P value
Eosinophil	0.5 (0.5–1.5)	5.25 (4–7.37)	25 (7–37.5)	0.003	< 0.0001	0.013
Neutrophil	0.5 (0.5–1.5)	8.25 (6.25–10.5)	4 (2.5–6)	< 0.0001	0.001	0.014
Total inflammatory cells	12.5 (10–20)	42.25 (34–54.5)	91.5 (68.5–129)	0.004	< 0.0001	0.012
CD4 T cell	1 (1–1)	2 (2–2.75)	4 (3–6)	0.016	< 0.0001	0.006
CD8 T cell	1 (1–1)	6.5 (4.25–8.75)	20 (6–29)	< 0.0001	< 0.0001	NS
Macrophage	1 (0–1)	6.5 (5–8)	12 (9–15)	0.003	< 0.0001	0.013
Mononuclear cells	8 (6–13)	13.5 (8.25–19.5)	30 (15–35)	NS	< 0.0001	0.010
Goblet cells	16.5 (10.5–21.5)	14 (10.25–17.5)	7.70 (5.5–13.5)	NS	0.004	0.026
Mucosal glands	22.5 (15.5–35)	19 (15.25–22.75)	14 (7.5–27.5)	NS	NS	NS

*NS* not significant

Values are expressed as median (IQR). Kruskal–Wallis test was used for unpaired comparisons. *Pv* < 0.05 was considered statistically significant

**Table 4:** Data representing eosinophil counts in CRSwNP patients

Patient group	Number	Age medians (ranges)	Female/male	Asthma	Eosinophil counts medians (IQR)
ECRS	16	35 (24–67)	6/10	5	35 (35–45)
N-ECRS	7	30 (14–38)	0/7	0	6 (5–7)

*ECRS* eosinophilic chronic rhinosinusitis, *N-ECRS* non-eosinophilic chronic rhinosinusitis

### Neutrophils were elevated in CRSsNP patients

**Fig. 4:**
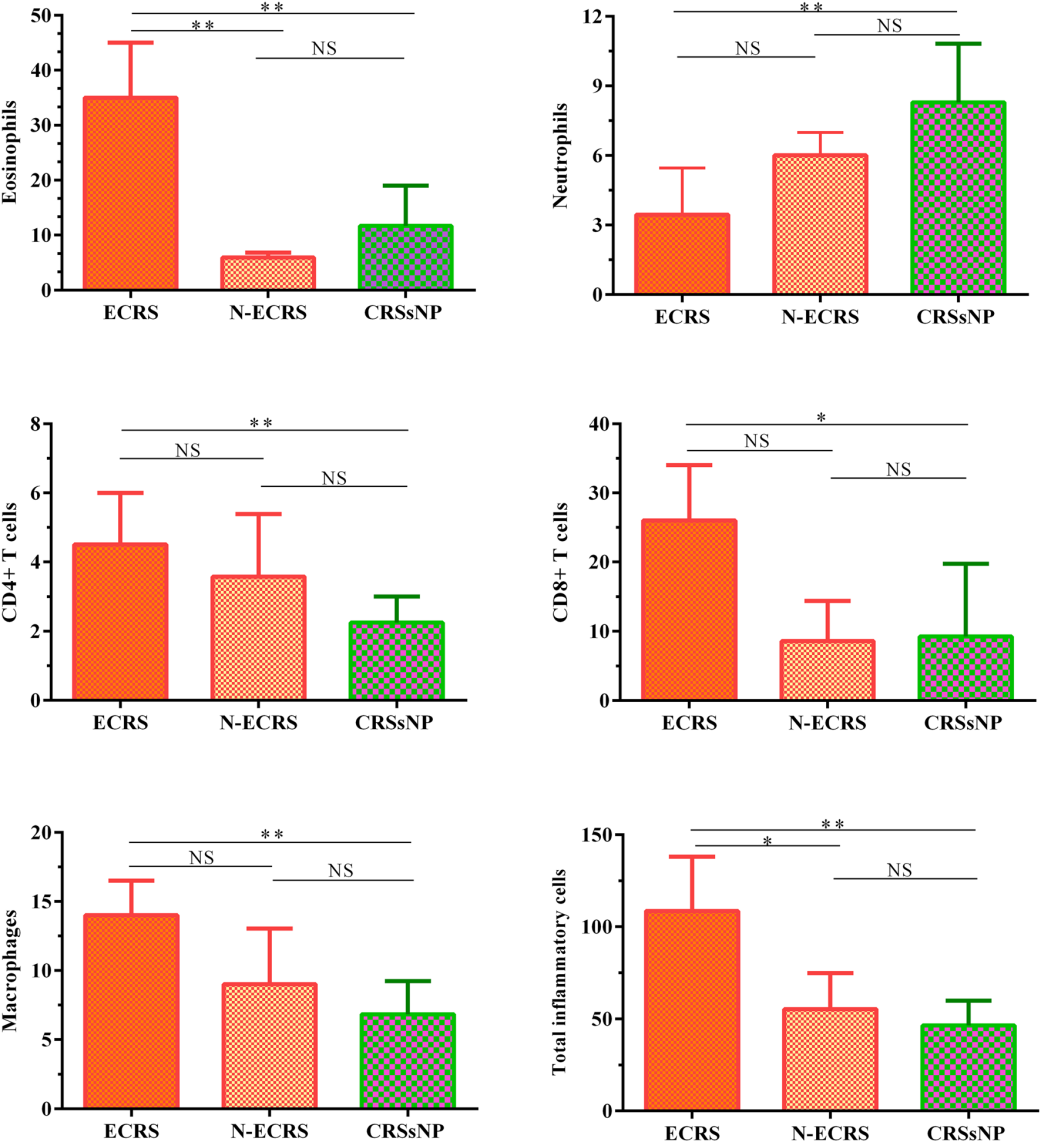
The mean numbers of eosinophils, neutrophils, CD4^+^ T cells, CD8^+^ T cells, macrophages, and total inflammatory cells in ECRS, N-ECRS, and CRSsNP groups. The Mann–Whitney U two-tailed test was used for between-group comparisons. *ECRS* eosinophilic chronic rhinosinusitis, *N-ECRS* non-eosinophilic chronic rhinosinusitis

The frequency of infiltrating neutrophils was significantly elevated in CRSwNP and CRSsNP groups in comparison to controls (*Pv* = 0.001 and *Pv* < 0.0001, respectively). However, the number of neutrophils was higher in CRSsNP group than CRSwNP group (*Pv* = 0.025) (Fig. 2, Table 3). ECRS patients had fewer neutrophils, while the N-ECRS patients presented significant neutrophil infiltration (Fig. 4, Table 5).

**Table 5:** Comparison of Immunohistochemistry results in eosinophilic and non-eosinophilic CRSwNP

	Eosinophilic CRSwNP	Non-eosinophilic CRSwNP	Eosinophilic vs Non-eosinophilic CRSwNP P value	Eosinophilic CRSwNP vs CRSsNP P value	Non-eosinophilic CRSwNP vs CRSsNP P value
Eosinophil	35 (23.13–45)	6 (5–7)	0.011	0.001	NS
Neutrophil	2.75 (1.625–5)	7.5 (5.5–9)	NS	0.001	NS
Total inflammatory cells	108.5 (91.5–138)	45.5 (40–77.5)	0.024	0.002	NS
CD4 T cell	4.5 (3–6)	3 (2–6)	NS	0.003	NS
CD8 T cell	26 (10–34)	6 (5–13)	NS	0.043	NS
Macrophage	14 (11–16.5)	8 (6–11)	NS	0.004	NS
Mononuclear cells	30 (15–40.63)	18.5 (8.25–19.5)	NS	0.006	NS
Goblet cells	9.25 (5.63–17.25)	7.5 (3.5–9.5)	NS	NS	0.028
Mucosal glands	13 (7.75–24.5)	22.5 (6–27.5)	NS	NS	NS

*NS* not significant

Values are expressed as median (IQR). Mann–Whitney U test was used for unpaired comparisons. *Pv* < 0.05 was considered statistically significant

### CD4^+^ T cells were elevated in CRSwNP patients, while CD8^+^ T cells and macrophages were increased in both CRSwNP and CRSsNP groups

CRSwNP group showed enhanced infiltration of CD4^+^ T cells into the sinonasal mucosa in comparison to CRSsNP group and controls (*Pv* < 0.0001 for CRSwNP vs. controls, *Pv* = 0.016 for CRSsNP vs. controls, and *Pv* = 0.006 for CRSsNP vs. CRSwNP) (Figs. 1 and 2, Table 3). In comparison to controls, both CRSwNP and CRSsNP groups indicated increased infiltration of CD8^+^ T cells into the sinonasal mucosa (both groups *Pv* < 0.0001). Meanwhile, there was no significant difference between CRSwNP and CRSsNP groups (Figs. 1, and 2, Table 3). Furthermore, the number of macrophages was significantly increased in CRS patients in comparison to controls (*Pv* < 0.0001 for CRSwNP vs. controls, *Pv* = 0.003 for CRSsNP vs. controls, and *Pv* = 0.013 for CRSsNP vs. CRSwNP) (Figs. 1 and 2, Table 3). We also found a significant difference in the number of macrophages between ECRS and CRSsNP patients (*Pv* = 0.004) (Fig. 4, Table 5).

### GATA3 was increased in CRSwNP patients, Ror-γt was increased in CRSsNP patients, and FoxP3 was decreased in both groups

**Fig. 5:**
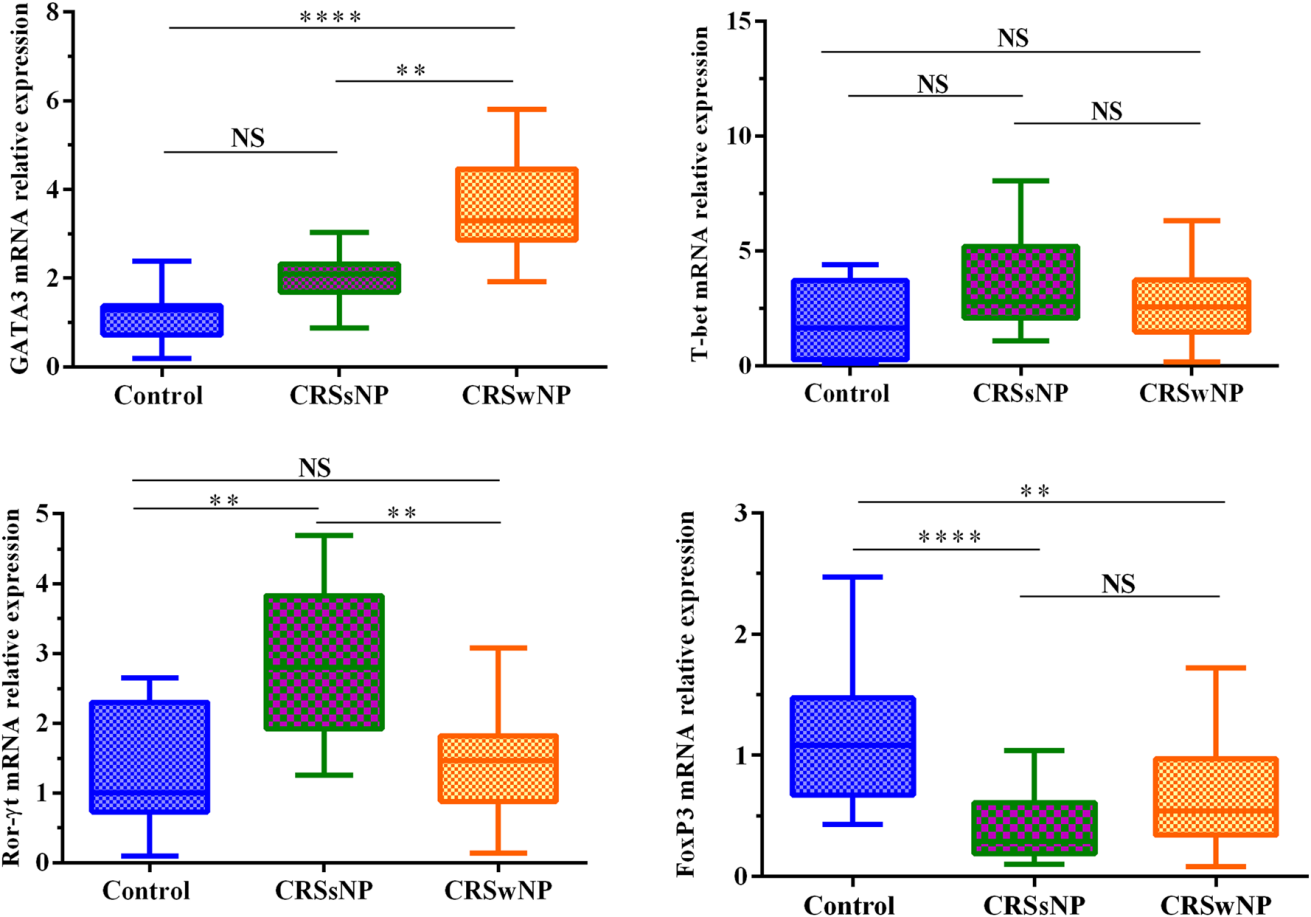
The expression levels of GATA3, T-bet, Ror-γt, and FoxP3 in controls, CRSsNP and CRSwNP patients. GATA3 was increased in CRSwNP patients, Ror-γt was increased in CRSsNP patients, and FoxP3 was decreased in CRS patients. No significant difference was reported for T-bet mRNA expression among three groups. The Kruskal–Wallis H test was used to assess significant intergroup variations

It was shown that mRNA expression of GATA3 was increased in CRSwNP patients compared with CRSsNP and control groups (*Pv* < 0.0001 for CRSwNP vs. controls, *Pv* = 0.001 for CRSsNP vs. controls) (Fig. 5). It represents Th2 dominance in CRSwNP patients. There was no significant difference in T-bet mRNA expression among three groups (Fig. 5). It was found that mRNA expression of Ror-γt was increased in CRSsNP patients compared with CRSwNP and control groups (*Pv* = 0.001 for CRSsNP vs. controls and also for CRSsNP vs. CRSwNP). No significant difference was found between CRSwNP and control groups (Fig. 5). It demonstrates Th17 dominance in CRSsNP patients. In comparison to healthy controls, both CRSwNP and CRSsNP groups showed decreased FoxP3 mRNA expression (*Pv* = 0.001 and *Pv* < 0.0001, respectively). There was no significant difference between CRSsNP and CRSwNP groups (Fig. 5).

## Discussion

Chronic rhinosinusitis (CRS) is an inflammatory condition affecting the sinonasal mucosa and T lymphocytes are important cells in the pathogenesis of CRS [25]. It is the first study conducted on the pattern of CD4^+^ and CD8^+^ T cells in Iranian CRS patients. In the current study, histological findings revealed that eosinophils and CD4^+^ T cells were elevated in CRSwNP patients. While neutrophils were increased in CRSsNP patients. On the other hand, CD8^+^ T cells and macrophages were increased in both CRSwNP and CRSsNP groups. In addition, real-time PCR results showed GATA3 was increased in CRSwNP patients, Ror-γt was increased in CRSsNP patients, and FoxP3 was decreased in CRSwNP and CRSsNP patients.

Here we show that the number of CD4^+^ cells is elevated in CRSwNP patients compared with controls, In consistent with previous studies [8, 22]. However, Cao et al. reported no significant difference between CRSwNP and CRSsNP patients as well as ECRS and N-ECRS groups [8]. In contrast, Baba et al. showed that CD4^+^ T cells were increased in N-ECRS vs. ECRS patients [21].

Although the importance of CD4^+^ T cells has been widely demonstrated in the pathogenesis of CRS, the function of CD8^+^ T cells in CRS development is not fully determined [26]. Numerous studies reported that the infiltration of CD8^+^ T cells was increased in nasal tissues of CRSwNP patients [8, 26]. Although we show that CD8^+^ T cells are predominant infiltrating T cells in the sinonasal mucosa of CRS patients, we found no significant difference between CRS patients. Our study also showed that there is no difference in the number of CD8^+^ T cells between ECRS and N-ECRS groups. However, a similar study showed that CD8^+^ T cell recruitment was significantly increased in adult Chinese CRS patients. In spite of our results, there was a significant difference between CRSwNP and CRSsNP groups, but similarly, they found no significant difference between ECRS and N-ECRS patients [8]. Bernstein et al. showed that the number of mucosal CD8^+^ T lymphocytes was increased in the ECRS and N-ECRS groups in comparison to the peripheral blood amount that this finding may demonstrate the local infiltration of CD8^+^ T cells and their possible roles in the progression of CRSwNP [27]. Another study conducted by Pant et al. showed that the percentage of mucosal CD8^+^ T cells in CRSwNP patients was higher than the peripheral blood of CRSsNP and controls [26]. Ma et al. evaluated the pattern of CD8^+^ T cell subsets who showed the percentage of Tc2 subset was positively correlated with eosinophil count, whereas the percentages of Tc1 and Tc17 subsets were positively correlated with neutrophil counts in CRSwNP patients [28].

Several contradictory explanations may explain higher CD8^+^ T cells over CD4^+^ T cells? 1-Tissue samples were collected from different anatomical regions which may be different in the pattern and frequency of inflammatory cells. Moreover, the simultaneous different Th cell patterns within a single tissue may be observed [9]. 2-Ethnical differences and life environment may affect CRS development [29]. 3-CD8^+^ T cells are potentially more resistant than CD4^+^ T cells against glucocorticoid therapy [8]. 4-Comorbidities such as asthma and atopy may play important roles in the severity and the pattern of infiltrating cells to the sinonasal tissues [30].

Our findings indicate that the number of macrophages is significantly increased in CRS patients. In consistent with our study, Cao et al. showed that the number of CD68^+^ cells was significantly higher in CRSwNP than CRSsNP patients, however, there was no significant difference between ECRS and N-ECRS groups [8]. Conversely, Van Zele et al. showed that although there was a trend toward a higher number of macrophages in CRS patients, no significant difference was detectable [31]. However, they didn’t exactly describe the macrophage phenotypes.

The current study shows that the frequency of eosinophils, neutrophils, and total inflammatory cells is increased in CRS patients in comparison to controls. Cao et al. reported similar results to our findings. They also showed a significant difference between ECRS vs N-ECRS and CRSsNP vs ECRS groups [8]. It was well-accepted that there is a direct association between eosinophil and neutrophil elevation in the amount of total inflammatory cells in CRSwNP and CRSsNP patients, respectively [8, 32]. We only observed a significant correlation between total inflammatory cells and eosinophils in CRSwNP patients. This finding is expected because major inflammatory cells are eosinophils in CRSwNP patients.

Previous studies have shown that CRSwNP patients are characterized by eosinophilic inflammation, Th2 dominance in western countries whereas Chinese or Korean CRSwNP patients are skewed toward neutrophilic inflammation and enhanced Th1/Th17 cell pattern, while Treg cells were significantly decreased [8, 29]. It has also been defined that the major characteristic of CRSsNP disease is increased number of Th1 cells in the sinonasal mucosa [31]. Our findings indicated that CRSwNP patients were Th2 dominant, while CRSsNP patients were Th17 dominant.

As Th2 cells promote eosinophilic inflammation and Th17 enhance neutrophilic inflammation; therefore, eosinophil recruitment to the sinonasal mucosa can contribute to the secretion, synthesis of specific granules, and release of lipid mediators as well as inflammatory cytokines and chemokines [1]. Through these inflammatory mediators, eosinophils can promote nasal polyp formation and progression. Although ECRS can be well-controlled via corticosteroid therapy, N-ECRS responds to a combination of macrolide therapy and surgical interventions [32].

In contrast, several studies reported that Th2 cells were reduced in CRSwNP patients [33, 34], whereas Zhang et al. indicated that there was no significant difference between CRSwNP and control groups [29]. Th17 responses are reported to be involved in the pathogenesis of multiple inflammatory diseases, including SLE and rheumatoid arthritis (RA). Some studies reported no significant difference in the number of Th17 cells between CRSwNP patients and controls [35, 36]. The results of different reports have shown that Th17 cells are elevated in CRSwNP patients in East Asian and Chinese populations in comparison to western societies [8, 37]. Wei et al. suggested a possible role of Th17 cells in an adult E-CRS Chinese patients [38]. In line with this study, Miljkovic et al. recently showed the frequency of Th17 cells was increased in CRSwNP patients [39]. Conversely, we reported that Th17 cells were increased in CRSsNP patients which it was the most interesting result in our research. This finding is contrary to the previous studies which have confirmed that Th1 cells are predominant T cells in CRSsNP patients [8, 31, 40]. However, the exact role of Th17 cells in white CRS patients is not fully determined [29, 35]. We propose that it may be associated with neutrophil accumulation in the sinonasal tissue of CRSsNP patients. Several other explanations for these discrepancies may be the ethnical differences, disease severity and previous therapeutic interventions such as glucocorticoid therapy or antibiotic consumption [33]. However, further investigations are required to fully elucidate this new finding. Finally, it is suggested to evaluate atopic status in CRSwNP patients and microbiological status in both groups, especially CRSsNP group. The major limitation of this study is its small sample size. Therefore, it is recommended that further research is necessary to be undertaken with larger sample population.

## Conclusion

Although conflicting results are reported on the role of T cells in CRS pathogenesis; our study showed that frequency of CD4^+^ and CD8^+^ T cells was increased in CRS patients. The findings of this research also provided insights for Th2 dominance in CRSwNP patients and Th17 dominance in CRSsNP patients. This study would result in several beneficial clinical outcomes, including targeted monoclonal therapies, introducing novel diagnostic and prognostic biomarkers, and reducing unwanted side effects and costs of medical treatments. Specific monoclonal antibodies can inhibit specific cytokines related to Th2 and Th17 cells in order to modulate the inflammatory conditions. Furthermore, by determining the miRNAs directing Th polarization toward different Th subsets, selected targeting therapies can be achievable. As the Eastern, Middle Eastern, Caucasian, and Western populations express heterogeneous inflammatory patterns in the composition of Th/Tc cells, underlying mechanisms in CRS may be different and subsequently, more histological investigations are needed to completely determine the function and the pattern of infiltrating T cells in CRS patients.

## Abbreviations

CRS: chronic rhinosinusitis
CRSsNP: chronic rhinosinusitis without nasal polyps
CRSwNP: chronic rhinosinusitis with nasal polyps
ECRS: eosinophilic chronic rhinosinusitis
FoxP3: forkhead box P3
HPF: high power field
N-ECRS: non-eosinophilic chronic rhinosinusitis
PBS: phosphate buffer saline
RORγt: retinoid acid-related orphan receptor γ
T-bet: T-box transcription Factor
